# Ca^2+^-Dependent Regulations and Signaling in Skeletal Muscle: From Electro-Mechanical Coupling to Adaptation

**DOI:** 10.3390/ijms16011066

**Published:** 2015-01-05

**Authors:** Sebastian Gehlert, Wilhelm Bloch, Frank Suhr

**Affiliations:** Institute of Cardiovascular Research and Sport Medicine, Department of Molecular and Cellular Sport Medcine, German Sport University Cologne, Am Sportpark Müngersdorf 6, Cologne 50933, Germany; E-Mail: w.bloch@dshs-koeln.de

**Keywords:** calcium, excitation-contraction coupling, ryanodine receptor, sarcoplasmic/endoplasmic reticulumcalcium ATPase, exercise, skeletal muscle, fiber type, protein turnover, calcineurin, peroxisome proliferator-activated receptor γ coactivator 1 α

## Abstract

Calcium (Ca^2+^) plays a pivotal role in almost all cellular processes and ensures the functionality of an organism. In skeletal muscle fibers, Ca^2+^ is critically involved in the innervation of skeletal muscle fibers that results in the exertion of an action potential along the muscle fiber membrane, the prerequisite for skeletal muscle contraction. Furthermore and among others, Ca^2+^ regulates also intracellular processes, such as myosin-actin cross bridging, protein synthesis, protein degradation and fiber type shifting by the control of Ca^2+^-sensitive proteases and transcription factors, as well as mitochondrial adaptations, plasticity and respiration. These data highlight the overwhelming significance of Ca^2+^ ions for the integrity of skeletal muscle tissue. In this review, we address the major functions of Ca^2+^ ions in adult muscle but also highlight recent findings of critical Ca^2+^-dependent mechanisms essential for skeletal muscle-regulation and maintenance.

## 1. Introduction

**Figure 1:**
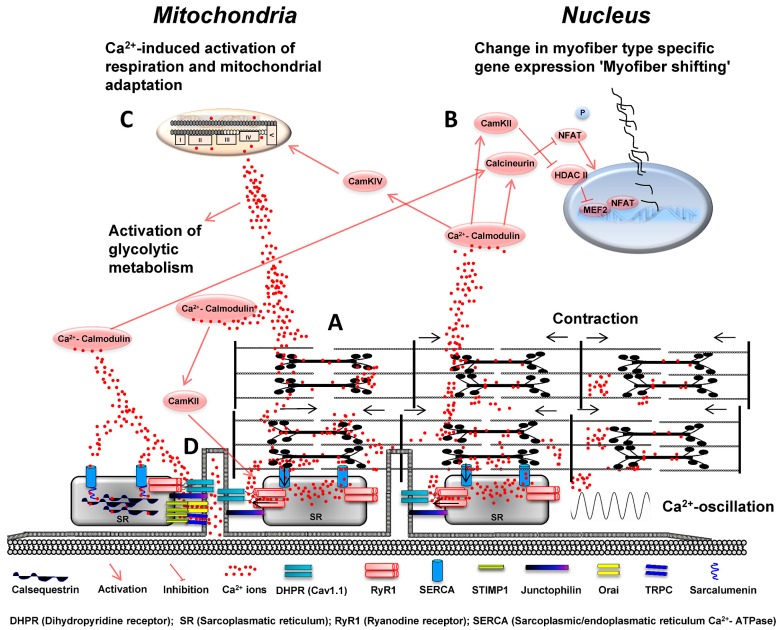
(**A**) Voltage-dependent activation of the dihidropyridine receptor (DHPR-Cav1.1) facilitates the release of Ca^2+^ ions out of the sarcoplasmatic reticulum (SR), which critically regulates skeletal muscle contraction. Reuptake of Ca^2+^ ions in the SR controls skeletal muscle relaxation and is mainly regulated by ATP-dependent sarcoplasmic/endoplasmic reticulum calcium ATPase pumps (SERCA1/2). Increased neuromuscular activity establishes an oscillating pattern of Ca^2+^ ion levels and causes elevated sarcoplasmic Ca^2+^ ion concentrations in the microenvironment of myofibrils; (**B**) Increasing levels of Ca^2+^ ions in the sarcoplasm bind to and activate calmodulin (CaM) which regulates activation of calcineurin and calmodulin kinase II and IV. Calmodulin kinase II (CaMKII) contributes to the phosphorylation of ryanodine receptor 1 (RyR1) which increases RyR1 channel activity and open probability. CaMKII further inhibits histone deacetylase II (HDACII) and increases nuclear abundance of myocyte enhancer factor 2 (MEF2). Calcineurin (CaN) dephosphorylates nuclear factor of activated T-cells (NFAT) hereby regulating its nuclear localization. NFAT and MEF2 facilitate the increased expression of “slow genes” coding protein isoforms of the oxidative fiber type; (**C**) CaMKIV increases the expression of mitochondrial genes, which contributes to mitochondrial adaptation. Free Ca^2+^ ions also directly stimulate or inhibit Ca^2+^ release via RyR1 in dependency of their luminal and sarcoplasmic Ca^2+^ concentration. Ca^2+^ ions further co-regulate the activation of energy metabolism by activating mitochondrial respiration and increasing the activity of glycolytic enzymes in sarcoplasm; and (**D**) store-operated calcium entry (SOCE) is regulated by stromal interaction molecule 1 (STIM1) which senses declined Ca^2+^ ion concentrations in the SR. Interaction of STIMP1 with Orai1 and canonical transient receptor potential channels (TRPC) leads to trans-sarcolemmal Ca^2+^ influx to increase intracellular Ca^2+^ levels upon declining Ca^2+^ content of the SR. Junctophilin maintains junctional triad integrity by overspanning the space between SR and plasma membrane and supports DHPR and RyR1 interaction. Ca^2+^ uptake and handling is enhanced by sarcalumenin which interacts with SERCA channels and calsequestrin.

Calcium ion Ca^2+^ distribution, movement and signaling are prerequisites for function and plasticity of skeletal muscle fibers. While the fast and acute oscillation of free Ca^2+^ levels in skeletal muscle is the major step in initiation of muscle contraction and relaxation, slower shifts of cytosolic Ca^2+^ levels are important contributors in the regulation of skeletal muscle plasticity by activation of specific signaling pathways such as the calmodulin/calcineurin signaling pathway (Figure 1).

The orchestra of Ca^2+^ signaling mechanisms in skeletal muscle determines a multitude of cellular processes. Already the initiation of muscle contraction at the neuromuscular junction is a Ca^2+^-dependent process at the motor endplate inducing a change in membrane polarization and a subsequent opening of l-type Ca^2+^ channels triggering the release of Ca^2+^ from the sarcoplasmatic reticulum (SR). This mechanism allows a distinct rise of cytosolic Ca^2+^ concentration that initiates actin/myosin interaction and movement of the myosin head. To facilitate the interplay of contraction and relaxation the SR is provided by several Ca^2+^ transport and binding molecules which are adjusted by a multitude of regulatory molecules. ATP production and hence energy supply of contracting muscle is also regulated by Ca^2+^-dependent enhancement of glycolytic enzyme activity and mitochondrial respiration. The high plasticity of skeletal muscle is enabled by Ca^2+^-dependent regulation of gene expression, translation and posttranslational processes including protein degradation. Ca^2+^ and, therefore, is involved in all processes required for hypertrophy, fiber type shift and maintenance of protein structures necessary for proper function of skeletal muscle. This review will highlight major Ca^2+^-dependent regulations for skeletal muscle function and remodeling.

## 2. Electromechanical Coupling in Skeletal Muscle

Skeletal muscle contraction is of vital importance for any directed movement of human beings. While in the earlier stages of human evolution this ensured the primary ability to fight, hunt and defend against opponents, it nowadays plays a more pivotal role for humans to engage in various kinds of sports and importantly, to maintain metabolic and musculoskeletal health. The directed modulation of force development during exercise is mediated by a complex neuromotoric recruitment pattern of distinct muscle fibers within skeletal muscles. In strict dependence of duration, force and speed of muscle contraction, the amount and type of recruited myofibers can be gradually adjusted by the nervous system to match the demands of the required skeletal muscle contractility [1,2]. However, the molecular basis for skeletal muscle contractility depends essentially on a mechanism commonly known as excitation-contraction coupling (ECC) [3]. This complex mechano-molecular regulation describes the interplay between an action potential which is induced by the nervous system and the resulting molecular interaction of myofilaments leading finally to mechanical force development and shortening of sarcomeres.

The multiple roles of Ca^2+^ ions in the regulation of acute contraction of skeletal muscle and the most important cellular structures which facilitate Ca^2+^ ion gradients and Ca^2+^ ion movement in skeletal muscle are highlighted and the feedback mechanisms that link initiation of contraction to sophisticated mechanisms of adaptation in exercising skeletal muscle is illustrated below.

## 3. Ca^2+^ Ions and the Regulation of Electro-Mechanical Coupling

### 3.1. Ca^2+^ Ions and the Initiation of Presynaptic Action Potentials

Ca^2+^ ions are vital for both the complex molecular interactions regulating the shortening of sarcomeres and for the prior electric activation along the motoric endplate that precedes the depolarization of the sarcolemma [4,5]. Upon arrival of an action potential, which is transduced via α-motoneurons of the central nervous system to the presynaptic neuron terminals, voltage dependent Ca^2+^ channels open and induce the Ca^2+^ ion influx from the extracellular space into the cytosol of the presynaptic neuron [6]. Ca^2+^ ions importantly initiate the interaction of acetylcholine containing vesicles with soluble NSF attachment protein receptors (SNARE) allowing these to fuse with the presynaptic membrane and to release acetylcholine into the synaptic cleft [4]. Nicotinic acetylcholine receptors (nAChRs), which also serve as ion channels, are located at the postjunctional folds of the sarcolemma and bind acetylcholine from the synaptic cleft [6,7]. This binding induces activation and opening of the ion channels leading to sodium (Na^+^) and potassium (K^+^) influx, which causes a local depolarization of the sarcolemma. Depending on the frequency of the incoming impulse pattern this local depolarization of the sarcolemma can be strong enough to cause a voltage-dependent opening of adjacent Na^+^ channels at the sarcolemma [8]. This transduces the local depolarization along the sarcolemma towards transverse tubular structures (T-tubuli) which are located in close vicinity to the adjacent terminal cisternae of the SR [9]. At this point, the local depolarization induces a voltage-dependent release of Ca^2+^ ions out of the SR which initiates cross-bridge cycling in myofibrillar compartments.

### 3.2. Ca^2+^ Storage and Localization in Skeletal Muscle

The majority of Ca^2+^ ions is mainly bound to Ca^2+^ binding proteins like calreticulin, parvalbumin, calsequestrin (calretinin) and sarcalumenin (SAR) in the SR under resting conditions [10,11]. While parvalbumin molecules are highly abundant in fast fibers of rodents and small mammals they are lacking in the myofibers of bigger animals and human skeletal muscle [12]. The SR is the essential intraluminal Ca^2+^ storage in skeletal muscle and critically involved in the contractile mechanism. It ensures the accumulation of high amounts of Ca^2+^ ions in the spatial vicinity of myofibers, which, upon the arrival of action potentials, are quickly released in the cytosol of myofibres facilitating the interaction of myosin and actin [13]. The SR constitutes a fine, membrane-surrounded tubular system tightly distributed between myofibres. Inside the SR, Ca^2+^ ions are stored via reversible binding to calreticulin, which is however abundant in a low density in skeletal muscle [14], and the highly abundant calsequestrin molecules (CASQ), which serve as high capacity-low affinity Ca^2+^ storage molecules [15]. Each molecule can bind up to 80 Ca^2+^ ions allowing storage of a high amount of Ca^2+^ in the SR upon release in the sarcoplasm of the myofiber. Two CASQ isoforms exist in skeletal muscle, calsequestrin 1 (CASQ1) in fast twitch type II fibers and calsequestrin 2 (CASQ2) in slow twitch or type I myofibers [16]. Both isoforms differ in their ability to bind and release Ca^2+^ upon stimulation and CASQ content is also higher in type II fibers than in type I fibers [15]. Hence, CASQ proteins are part of a network of highly specific Ca^2+^ handling proteins that differ between type I and II myofibers [16] and which importantly also contribute to the distinct contractile characteristics of myofiber types. SAR is abundant in cardiac and skeletal muscle, located at the longitudinal tubuli and the terminal cisternae [17] and similarly expressed in type I and II myofibres. SAR has multiple functions in skeletal muscle Ca^2+^ handling, and like CASQ, also serves as a Ca^2+^ buffering molecule in the SR [17]. However, SAR has been shown to be phosphorylated and to modulate the activity of the junctional ryanodine receptor 1 (RyR1) channel complex and thereby also Ca^2+^ release characteristics [18]. SAR supports proper Ca^2+^ reuptake in skeletal muscle as it has been shown to be co-localized and interacting with sarcoplasmic/endoplasmic reticulum calcium ATPase 1 (SERCA1) [19] acting as a linker protein between Ca^2+^ uptake units, storage proteins and the Ca^2+^ release units of the SR. Hence, SAR knockout mice show a reduced relaxation time of skeletal muscle despite normal force generation emphasizing a dominant role of SAR in maintaining Ca^2+^ uptake and buffering [20]. Importantly, it was demonstrated recently that SAR knockout mice bear a potential for decreased muscle fatigue *in vivo* and *in vitro* [21], which was mechanistically accompanied by upregulated genes, such as mitsugumin 29. Therefore, the authors concluded that a deletion of SAR results in improved function store-operated calcium entry (SOCE) (see Section 3.3), consequently compensating for the potential of increased muscle fatigability. In resting skeletal muscle, Ca^2+^ concentrations are reported to be between 20 and 50 nM [22,23], though others reported values ranging from 100 to 250 nM [24,25]. The maintenance of this concentration is ensured primarily by the retention of Ca^2+^ ions in the SR via binding to CASQ1 and 2, the active reuptake from the cytosol in the SR via ATP-dependent Ca^2+^ pumps (SERCA1 and 2) [26] or across the sarcolemma via the Na^+^-Ca^2+^ antiporter (NCX1) [27]. Interestingly, the cytosolic free Ca^2+^ level is higher in type I than type II fibers already under resting conditions. As described later, this contributes to the structural differences in the composition of fiber types due to differential rates of Ca^2+^-dependent signaling at rest and increased neuromuscular activity.

### 3.3. Regulation of Ca^2+^ Homeostasis via Store-Operated Calcium Entry (SOCE) and Molecules Regulating SR Integrity

The maintenance of the adequate Ca^2+^ concentration in extracellular and intracellular compartments of skeletal muscle is of crucial necessity for sustained contractility of skeletal muscle fibers. Therefore the facilitation of Ca^2+^ ion movements in skeletal muscle requires a complex interaction of differentially regulated subsystems. Besides the fast and voltage-dependent activation of the initial Ca^2+^ influx from of the SR via RyR1, the SOCE has a slower kinetic [28], but offers a further and important mechanism that contributes to maintain proper contractile function by facilitating trans-sarcolemmal Ca^2+^ influx when SR calcium stores begin to be depleted [29]. SOCE depends primarily on the molecular interaction between stromal interaction molecules (STIM1), calcium permeable Orai1 channels (Orai1) and canonical transient receptor potential channels (TRPC) in skeletal muscle [30,31]. STIM1 is a single-pass transmembrane protein in the SR-membrane and its EF-hand domain is located in the lumen of the SR where it is able to sense changes in Ca^2+^ store content. The coiled-coil domains located in the cytosolic part are believed to interact with Orai1, a tetrameric ion channel complex which facilitates increased Ca^2+^ ion conductance across the sarcolemma upon declined SR Ca^2+^ content [29]. A similar interaction between STIM1 and TRPC channels and also between TRPC, RyR1 and Ourai1 were determined [32,33] and are believed to contribute to SOCE, although the exact mechanisms are still unresolved. The main tasks for SOCE relies on the increased delivery of Ca^2+^ in the cytosol of the myofiber hereby supporting the requirement of high Ca^2+^ gradients under repeated contractions and to enhance Ca^2+^ sensitivity of myofilaments (see Section 3.8).

The importance of SOCE for sustained contractility of skeletal muscle has become evident by pharmacological inhibition of SOCE, which exerts significantly declined skeletal muscle contractility in young but not aged muscle [34]. The latter finding attributed SOCE as a likely mechanism that contributes to explain the generally reduced skeletal muscle contractility in aged skeletal muscle. Importantly, skeletal muscle from STIM1-deficient mice showed reduced expression of contractile proteins and SERCA1 which highlights also the link between SOCE-induced Ca^2+^ entry and gene expression. Ca^2+^-induced signaling (see Section 5, Section 6 and Section 7) in skeletal muscle is critically dependent on the kinetics of Ca^2+^ transients between the extracellular and intracellular compartments and is a prominent feedback mechanism for muscle adaptation [35]. SOCE has been shown to contribute significantly to enhanced gene expression via nuclear factor of activated T-cells (NFAT) [30,36] and, hereby, also expands the network of Ca^2+^-dependent feedback mechanisms which link neuromuscular activity and Ca^2+^ ion movement with long term muscle remodeling (see following sections).

Skeletal muscle further requires a variety of molecules that coordinate the integrity of Ca^2+^ handling proteins during development as well as ensure proper Ca^2+^ handling in adult skeletal muscle and which are indirectly associated in the regulation of voltage-induced Ca^2+^ release via RyR1 or also SOCE. Amongst these, junctophilin (JP) and mitsugumin (MG) play prominent roles in facilitating the complex interplay of structures involved in Ca^2+^ handling in skeletal muscle [37,38]. JP proteins belong to a family of junctional membrane protein complexes overspanning the space between plasma membrane and SR [39]. JP-1 and JP-2 are both expressed in skeletal muscle and contribute to the formation of junctional membrane complexes and the physical coupling between Cav1.1 and RyR1 to support their interaction and Ca^2+^ release [40]. At least JP-1 possesses important roles in maintaining the structural integrity of triad junctions as JP-1 knockout mice display structural abnormal triad junctions, altered sensitivity towards Ca^2+^ and reduced force generation capacity of skeletal muscle [41]. In this context, MG proteins also belong to essential membrane proteins of the triads that serve important and similar roles like JP-1 and JP-2 [42]. It has been reported that the knockout of MG29 in mice, e.g., leads to abnormal formations of SR networks during development which are maintained also in adult muscle [43]. Furthermore, knockout of MG53 was reported to attenuate SERCA1 activity in rabbit skeletal muscle only under conditons of micromolar Ca^2+^, hence, emphasizing a functional role for MG53 to control Ca^2+^ handling especially during muscle contractions [44].

### 3.4. Regulation of Ca^2+^ Release via Voltage and Coupled Gating

As the evoked depolarization wave runs along the sarcolemma it finally induces the voltage dependent activation and conformational change of the subunits of the l-type Ca^2+^ channel or dihydropyridine receptor (DHPR or Cav 1.1) [45]. The DHPR itself is associated via a protein interaction with the ryanodine receptor 1 (RyR1). RyR1 is the biggest Ca^2+^ ion channel in organisms and its name is derived from the plant alkaloid ryanodine which reversibly binds to the ryanodine receptor and, at micromolar concentrations, induces the full opening of RyR1 channels [46]. This exerts a massive Ca^2+^ influx into myofibers of skeletal muscle and muscular paralysis due to sustained contraction of fibers. This mechanism emphasizes the importance of Ca^2+^ release out of the SR for skeletal muscle contraction but also highlights the necessity for a precisely regulated control of RyR1 channel activity in skeletal muscle [47]. The RyR1 is a giant homotetrameric Ca^2+^ channel complex composed of four identical subunits each associated with calstabin molecules (also known as FKBP12 or FK506 binding protein) [48]. The channel complex is located in the SR membrane with its cytoplasmatic domain interacting with the DHPR, overspanning and connecting the ultrastructural space between the t-tubular and SR membrane and the terminal cisternae of the SR, respectively [9]. The luminal domain of RyR1 is located inside the SR and interacts with CASQ but also with triadin and junctin (see Section 3.5), which serve as interconnectors between CASQ, RyR1 and the SR membrane [49]. RyR1 channel isoforms are identical within type I and II myofibers, however, type II myofibers possess a higher density of RyR1 channels than type I fibers [50]. This circumstance is comprehensible as type II myofibers require higher Ca^2+^ gradients for the initiation of skeletal muscle contraction [51]. Under basal conditions, the conformational state of RyR1 leaves the channel usually in a closed condition, hence not allowing higher amounts of Ca^2+^ ions to leave the SR without appropriate signaling [9]. The activation of the DHPR via voltage-dependent depolarization of the sarcolemma induces a mechanical protein interaction of the DHPR with RyR1 [13], the dissociation of calstabin molecules (FKBP12) from the RyR1 subunits and the opening of the channel complex. As a primary consequence of the voltage-dependent DPHR opening of RyR1 also known as “voltage-gating”, Ca^2+^ ions are rapidly released from the SR into the sarcoplasma of the myofiber [52]. Voltage-dependent opening of the RyR1 channel induces the rapid increase of the myofibrillar Ca^2+^ concentrations from around 20–250 nM [23,25] up to 100-fold within myofibrillar compartments. However, voltage-dependent gating alone does not represent the only trigger to induce a massive change in Ca^2+^ concentrations upon action potentials. Only around 50% of all RyR1 channels are directly interacting with DHPR. The remaining channels are located as highly organized clusters around their DHPR interacting neighbors and are attached to each other at the corners of the RyR1. This molecular interaction ensures that neighbored RyR1 channels can be opened without DHPR-dependent activation by a molecular interaction between adjacent RyR1 channels and by ensuring RyR1 channels amplify their Ca^2+^ release signal. Clustered activation of RyR1 channels is also termed as “coupled gating” [53]. This mechanism is essential to create waves of high Ca^2+^ transients in myofibers, the so called Ca^2+^ sparks [54]. Ca^2+^ sparks in skeletal muscle constitute highly localized Ca^2+^ concentration gradients ensuring Ca^2+^ ions to effectively initiate the molecular interaction between force generating filaments in sarcomeres.

### 3.5. Regulation of RyR1 Activity and Ca^2+^ Release via Interacting Molecules and Channel Modification

The complex structure of the large RyR1 channel complex ensures its sophisticated function in regulating skeletal muscle contractility. RyR1 channels can interact with a row of molecules that coregulate, fine-tune or inhibit the activity of RyR1 and hence the generation of Ca^2+^ gradients in skeletal muscle [55,56]. RyR1 can be subjected to diverse posttranslational modifications (e.g., phosphorylation and nitrosylation) on several residues serving as a feedback system of the local mechano-metabolic environment of the RyR1 channel [57]. The exact and entire mechanisms by which Ca^2+^ release from RyR1 is regulated are highly complex and shall not be discussed in detail in this review. However, the most important interactions and modifications of the RyR1 channel will be highlighted.

In the lumen of the SR, CASQ is the main Ca^2+^ binding protein. However, despite the important role in Ca^2+^ binding, CASQ offers also the ability to influence RyR1 channel activity by mechanical interaction [58]. It has been suggested that dependent on luminal Ca^2+^ content, conformational changes of CASQ in the SR membrane can occur which modulates the molecular interaction with RyR1 in dependence of the luminar Ca^2+^ concentration and by its association with the SR membrane. It has been further shown that CASQ can be phosphorylated and that RyR1 open channel probability is increased in dependence of increased CASQ phosphorylation. Junctin and triadin are important interconnecting proteins between CASQ and RyR1. RyR1 activity is regulated by molecular interaction with Ca^2+^-dependent CASQ that is anchored to triadin and also the SR membrane [49]. Junctin, with a high sequence homology to triadin, has been found in junctional membranes and seems to serve similar roles as triadin in the SR. Smaller molecules like ATP and Mg^2+^ regulate Ca^2+^ release by binding to the inhibitory I-sites and activating A-sites of the RyR1 complex which modulate channel activity [59]. Mg^2+^ binds preferentially to the inhibitory I-site but also the activating A-site of the RyR1 complex, importantly by competing with Ca^2+^ ions, which bind to this site and serve as strong activators of RyR1. Under resting conditions Mg^2+^ ions act as strong inhibitors of RyR1 channel activity while ATP in contrast increases RyR1 channel activity and Ca^2+^ release. Mg^2+^ ions are often bound to ATP forming an ATP-Mg^2+^ complex. Under conditions of exercise, increasing ATP turnover in skeletal muscle induces the decline in ATP levels and an increase in free Mg^2+^. This change in metabolic environment acts as a feedback system inducing decreased Ca^2+^ release via RyR1 channel inhibition by Mg^2+^ ions. Besides the acute modulation of RyR1 activity and therefore Ca^2+^ release via changed sarcoplasmatic environment, there are further modifications known that are reversible, more stable and hence provide a timely extended modulation of RyR1 channel activity. For example, chronic adrenergic stimulation has been shown to induce phosphorylation of RyR2 via protein kinase A (PKA) in myocardium but also RyR1 in skeletal muscle [60]. Phosphorylation of RyR1 has been associated with increased fatigability of skeletal muscle due to leaky RyR1 channels and dissociation of FKBP12 [48,57]. In contrast, PKA-dependent phosphorylation of RyR1 has also been shown to be essential for force development of contracting skeletal muscle [61].

Nitric oxide synthase (iNOS, nNOS) is another molecule owing important roles in modulating Ca^2+^ release via RyR1 [62,63]. Nitric oxide (NO) release via neuronal NOS (nNOS) and inducible NOS (iNOS) is dependent on mechanical strain of skeletal muscle at costameres but also on Ca^2+^ [64]. Nitrosylation of RyR1 has also been shown to occur in contracting skeletal muscle decreasing RyR1 activity via NO-affected binding and decreased channel conductance [57]. As a consequence, NO abundance can reduce force generation of skeletal muscle within contracting myofibers. As each of the four RyR1 subunits has several modification sites [47], it can be hypothesized that phosphorylation and nitrosylation of RyR1, in interplay with other regulators [65] and in dependency on the chemical nature of the modification, may finally regulate increased or decreased skeletal muscle contractility.

### 3.6. Modulation of RyR1-Induced Ca^2+^ Release via Ca^2+^ Ions and Calmodulin

**Figure 2:**
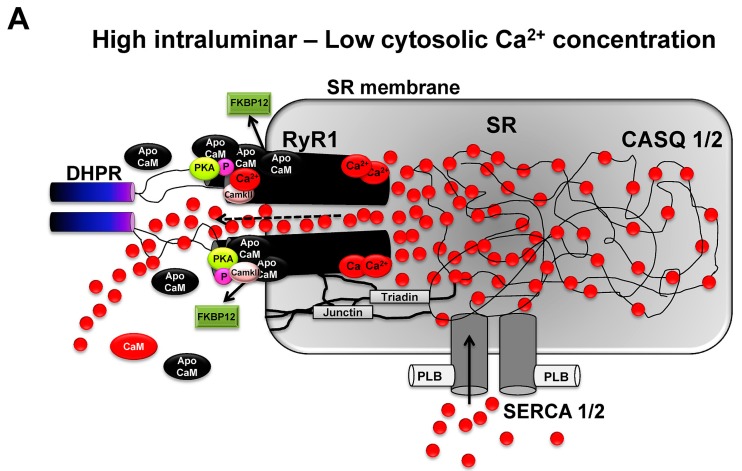

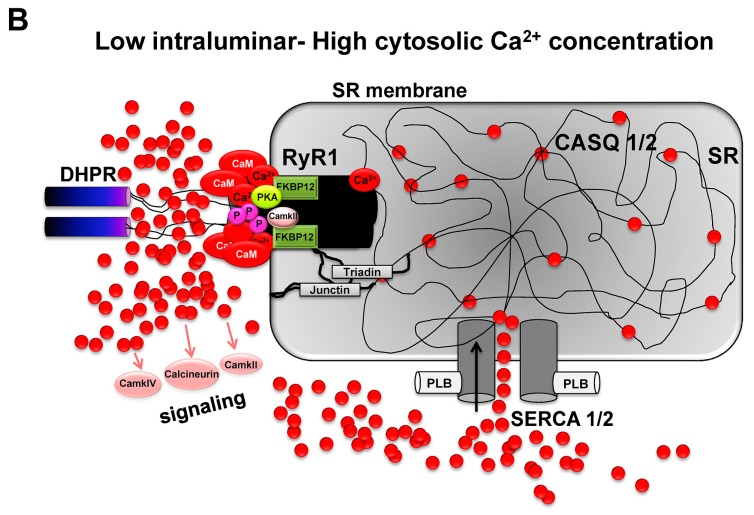
RyR1 channels induce rapid and high gradients in Ca^2+^ ion concentration between luminal compartments of the SR and surrounding sarcoplasm of myofbrills. (**A**) RyR1 opening is primarily controlled by the voltage dependent activation of DHPR which mechanically interacts with RyR1 and regulates channel opening (voltage gating). Further and adjacent RyR1 channels are opened by voltage-independent RyR1-RyR1 interactions (coupled gating) which create locally high Ca^2+^ ion gradients. On the luminar side of the SR, RyR1 channel opening is supported by the combined mechanical interaction with triadin, junctin and the SR membrane. High Ca^2+^ ion concentrations in the SR further supports channel open probability. On the sarcoplasmic side and at low Ca^2+^ concentrations, high amounts of Ca^2+^ unbound apoCaM supports increased open probability and activity of RyR1 while Ca^2+^-activated CaM inhibits RyR1. PKA and CaMKII have binding sites on RyR1 subunits and are able to phosphorylate RyR1 and modulate channel activity; and (**B**) Upon elevation of sarcoplasmic Ca^2+^ levels due to RyR1 channel opening, decreased Ca^2+^ ion levels in SR inhibit RyR1 channel activity. Sarcoplasmic Ca^2+^ levels increase CaM levels which activate CaMKII, IV and calcineurin. CaM inhibits RyR1 channel activity on the sarcoplasmic side of RyR1 while CaMKII binds to and phosphorylates RyR1. Hyperphosphorylation of RyR1 via PKA and CaMKII may lead to increased dissociation of FKBP12, higher open probability of RyR1 channels and decreased contractility of skeletal muscle under resting conditions. Increased SERCA activity facilitates rapid reuptake of Ca^2+^ ions into the SR.

Ca^2+^ ions themselves have a high capability in modulating RyR1 channel activity and, thus, offer an important role in the modulation of their own release via modulation of RyR1 [66]. Ca^2+^-dependent modulation of the RyR1 channel occurs in different ways. As a direct effect, Ca^2+^ binds either to the high affinity and activating A-site of RyR1, which increases channel activity or the inhibitory I-site which then decreases RyR1 channel conductance. RyR1 is primarily activated at low Ca^2+^ concentration (0.5 µM) and inhibited by elevated concentrations of Ca^2+^ (0.15 mM). Hence, RyR1-induced Ca^2+^ release is directly affected by Ca^2+^ ions via a positive and negative feedback regulation. By this, RyR1 channel conductance is regulated by two distinct Ca^2+^ binding sites on the cytoplasmic domain of the RyR1, controlling and sensing luminal Ca^2+^ flux into modulated RyR1 channel conductance. Typically, at low Ca^2+^ concentration, CaM is preferentially not activated (apoCaM), whereas elevated Ca^2+^ concentration binds to and activates calmodulin (Ca^2+^-CaM) [67,68]. ApoCaM binds to and activates RyR1, whereas CaM inhibits RyR1 channel conductance. Activated CaM can further activate calmodulin kinase II (CaMKII) which, when activated, phosphorylates RyR1 [9,55]. This can contribute to the discussed increase or decrease in skeletal muscle contractility, which has been observed in response to the phosphorylation of RyR1 channel subunits. Figure 2 illustrates the activity of RyR1 channels upon inhibition and activation by interaction with the most important regulators.

### 3.7. Ca^2+^-Dependent Control of Crossbridge Cycling and Force Generation

The primary force generating mechanism (≈5 pN per sarcomere) depends on the regulated molecular interaction between myosin and actin filaments within sarcomeres. Myosin molecules are composed of two heavy chains (MHC) containing the ATP hydrolyzing head region which binds to actin. Each myosin heavy chain is further associated with two myosin light chains (MLC) binding the neck region of MHCs. Under conditions of low Ca^2+^ concentrations, the binding sites of the myosin heads on actin filaments are blocked by tropomyosin molecules. Tropomyosin molecules are tightly associated with a troponin complex that contains the subunits troponin T which associates the troponin complex to tropomyosin, the Ca^2+^-binding and regulatory subunit (troponin C) and troponin I as the inhibitory subunit which blocks myosin binding sites on the actin filaments [69]. Upon Ca^2+^ release via RyR1, two Ca^2+^ ions bind rapidly to troponin C leading to a conformational change within the troponin complex. This induces a release from the inhibitory troponin I subunit from actin, allowing myosin head regions to bind to actin binding sites [70]. At this moment, myosin heads follow the transition from a weak binding state to a strong binding state at which ATP is fully hydrolyzed and inorganic phosphate (Pi) is released. The swinging lever arm action of the myosin head generates the relative movement of thin and thick filaments to each other which, consequently, shortens the sarcomere and generates force. This strong binding state of myosin heads persist until one molecule of ATP is replacing ADP which is still bound to MHC heads [3]. The rate at which the strong binding of myosin head to actin occurs is critically dependent on Ca^2+^ ions bound to troponin C [70]. Thus, myofilament interaction is facilitated by the controlled abundance of Ca^2+^ ions and skeletal muscle force is directly dependent on the Ca^2+^ concentration [71]. No nearby force is generated at very low Ca^2+^ concentration, whereas steadily increasing Ca^2+^ concentrations lead to increased force development of skeletal muscle. This dependency of force development on Ca^2+^ concentration, however, offers a saturation effect where at some point increasing Ca^2+^ levels do not result in further force development of skeletal muscle. The reason for this is that, under rising calcium concentrations, all Ca^2+^-binding sites on actin are eventually occupied. Although a high Ca^2+^ ion concentration in the cytosol of myofibres is essential for contractility and force development of skeletal muscle, Ca^2+^ can also lead to declined contractility and muscular fatigue. Besides chronically elevated Ca^2+^ levels in myofibrillar compartments e.g., due to leaky RyR1 channels (see Section 3.5), under conditions of severe and repeated muscle contractions, also increased abundance of inorganic phosphate (P_i_) and Ca^2+^ ions have been shown to build Ca^2+^-P_i_ precipitates [72,73,74] which blunt skeletal muscle contractility.

### 3.8. Ca^2+^ Ions and the Regulation of Force Development via Modulation of Regulatory Light Chain Phosphorylation

Force development of myofilaments can further be influenced by myosin light chains (MLC) [75,76]. Each myosin heavy chain molecule is surrounded by two MLCs of which one possesses the role of a regulatory light chain (RLC) [22]. RLCs can be phosphorylated by myosin light chain kinase (MLCK) [77]. Ca^2+^ ions mediate this process as they bind to and activate CaM which then activates myosin light chain kinase (MLCK). Hence, under resting conditions and when Ca^2+^ ion abundance in myofibrillar compartments is low, RLCs remain mainly unphosphorylated. However, under repetitive contractions the phosphorylation state of RLCs rises and is accompanied by an increase in post-tetanic force of myofibers [78]. This phenomenon is described as post-tetanic twitch potentiation of myofibers and describes the fact that within one second after maximal tetanic activation, a second twitch exerts a higher force than the prior contraction [78]. This staircase effect of increasing contraction force is believed to be exerted to a high extent by increasing RLC phosphorylation which increases the Ca^2+^ sensitivity of the contractile apparatus. The molecular reason for this phenomenon can be explained by changed molecular structure of RLCs which alters also the molecular properties of MHCs during cross-bridge cycling [79]. Myosin heads are in the unphosphorylated state of RLCs tightly bound to the backbone of the thick myosin filament whereas the organization of myosin heads results in a more loosened and disorganized state when RLCs become phosphorylated. This was attributed to a higher flexibility of the myosin head and increased probability to interact with actin due to a closer proximity of the myosin heads. Interestingly, RLC phosphorylation persists for a time also upon increasing fatigue of skeletal muscle. This ensures that rising CaM levels which inhibit Ca^2+^ release by RyR1 are also accompanied by a higher Ca^2+^ sensitivity of the myofibrillar apparatus which contributes to sustained contractility of sarcomeres under these conditions [3]. Hereby, Ca^2+^ ions indirectly serve as regulators that help sparing of ATP by changing the molecular-mechanic properties of MHCs under conditions of increasing metabolic fatigue.

## 4. Ca^2+^ Ions and the Regulation of Skeletal Muscle Relaxation

### Regulation of Ca^2+^ Reuptake into the SR

During exercise, directed Ca^2+^ ion release is under the control of RyR1 and the formerly mentioned regulators that adapt RyR1 activity to match the demands of the required contractility. However, under these conditions, Ca^2+^ ions have to be continuously removed after the absence of action potentials to ensure relaxation of sarcomeres and to prepare the next directed contraction [80]. Relaxation of skeletal muscle occurs when the intracellular Ca^2+^ concentration returns to nearby resting levels allowing troponin to return in the closed position in which binding of myosin heads to actin is blocked [3]. Ca^2+^ removal is under the main control of three distinct ion channels named ATP-dependent SERCA [26], plasma membrane ATPase (PMCA) and the Na^+^/Ca^2+^-exchanger (NCX1) [27]. SERCA channels are 100 kDa integral membrane proteins that regulate myofibrillar Ca^2+^ removal by pumping Ca^2+^ ions back in the SR under ATP usage. This process occurs under a complex, mutual regulation of Ca^2+^ binding and ATP hydrolysis along substructures of the channel structure which forms a globular lobe protruding into the cytosol [5]. Although about 10 isoforms exist in vertebrates, SERCA1a and SERCA2a channels are the major isoforms in adult human skeletal muscle [81]. While SERCA1 is the isoform in fast twitch type II myofibres, SERCA2 is expressed in type I myofibers. SERCA density and also Ca^2+^ uptake is five- to seven-fold higher in type II than type I myofibers contributing to the fact that also the SR is more developed in fast fibers [22]. Additionally, SERCA isoforms differ in their responsiveness to ADP which inhibits its activity and Ca^2+^-leak properties [82]. Both mechanisms are important to withstand muscular fatigue, as type I slow fibers show a smaller responsiveness to ADP and reduced Ca^2+^-leakage when compared to more fatigable type II fibers [82]. SERCA pump activity can further be inhibited via lowering their Ca^2+^-affinity by extraluminal proteins phospholamban (PLB) which is mainly responsible for SERCA2 inhibition. Sarcolipin (SLN) interacts with SERCA1 in fast fibers [83]. However, in a recent paper, the co-expression of PLN and SLN in slow and fast twitch human skeletal muscle fibers was demonstrated [84] and it was concluded that the abundance of both proteins can act by enabling a super-inhibition of SERCA [85]. PLB itself can be inhibited from its inhibiting function of SERCA2 by phosphorylation [86]. In the t-tubule fraction of slow and fast contracting myofibres, PMCAs [87] are highly abundant in close vicinity to the DHPRs and contribute to ATP-dependent Ca^2+^ removal [27]. It is believed that especially in the gap between t-tubules and terminal cisternae of the SR, Ca^2+^ concentration would be extremely high upon RyR1-induced Ca^2+^ release, which would inhibit RyR1 activity. Hence, the accumulation of PMCAs in this environment can contribute to sustained contractility of myofibers by pumping Ca^2+^ ions in the lumen of the t-tubuli. NCX channel isoforms (NCX1-3) also contribute to the trans-sarcolemmal calcium flux especially in slow type I myofibers, although to a minor extent. Differences in isoform activity and their relative contribution to Ca^2+^ efflux are under investigation.

## 5. Ca^2+^ Ions and the Regulation of Energy Metabolism

### 5.1. Ca^2+^ Ions and the Regulation of Glycolysis

ATP is required for Ca^2+^-handling during contraction as it ensures Myosin-ATPase, SERCA1 and 2, and other ATP-dependent ion channels to regulate cross-bridge cycling and relaxation of myofibers by maintenance of the correct Ca^2+^ distribution within subcellular compartments of skeletal muscle [3]. To ensure sustained contractility of skeletal muscle, the generation of ATP has to match the demands during contraction. A major metabolic pathway in skeletal muscle that provides a high amount of ATP generation per time is the anaerobic glycolysis which converts one molecule glucose to two molecules pyruvate or lactate and two molecules ATP and, in case of glycogen utilization, three molecules ATP per molecule glycogen [88]. The regulation of glucose or glycogen breakdown is relatively short-stepped and requires fewer enzymatic driven reactions when compared to aerobic oxidation (e.g., free fatty acids). Ca^2+^ ions contribute to the regulation of glycolysis as they affect the enzymatic speed of crucial enzymes of the glycolysis [89]. Glycogen degradation to pyruvate requires glycogenphosphorylase (GPL) which converts one molecule of glycogen to glucose-1-phosphate and primes its further degradation via glycolysis to lactate. The phosphorylation and activation of GPL depends on the activity of the enzyme phosphorylase kinase (PhK). Years ago, it was demonstrated that the important Ca^2+^-binding molecules CaM and troponin C regulate the activity of PhK in interplay with Ca^2+^ ions and the phosphorylation by PKA [90]. PhK in its unphosphorylated form (PhK b) form is relatively inactive when Ca^2+^ concentration is low. PKA can phosphorylate PLK on its β-subunit transforming it to its active form (PhK a). However, dependent on Ca^2+^ concentration, Ca^2+^ ions bind to the δ-subunit of PhK which has a high sequence homology to calmodulin. This mediates an important step in the activation of PhK, however, the additional interaction of PhK with sarcomeric troponin-c seems to be required for the further activation of PhK. The muscle specific isoform of phosphofructokinase (PFK-M) is the most important pacemaker of glycolysis rate. It catalyzes the reaction from fructose 6 phosphate to fructose 1–6 bisphosphate which together with AMP allosterically regulate PFK activity in contracting muscle. Ca^2+^ ions are able to modulate PFK activity by the Ca^2+^-dependent activation of CaM which interacts with PFK [91]. PFK monomers have two binding sites for CaM. CaM binding to the high affinity site of PFK forms the generation of stable PFK dimers which exhibit increased catalytic activity of PFK, in part preventing allosteric inhibition of the enzyme, e.g., by ATP, citrate and lactate. The formerly described regulations facilitate the full activation of PhK and contribute to increased PFK activity via increased abundance of Ca^2+^. Hence, these Ca^2+^-dependent mechanisms serve as an important contribution to coordinate the onset of muscle contractions with mechanisms that augment energy metabolism in working muscle.

### 5.2. Ca^2+^ Ion-Dependent Regulation of Mitochondrial Function

Ca^2+^ influx into mitochondria has been shown to result in increased energy conversion potential which is necessary in the maintenance of energetic homeostasis in contracting muscle [92]. The Ca^2+^-transmitted to the mitochondria elevates matrix Ca^2+^ concentration, which activate Ca^2+^-sensitive dehydrogenase activities, leading to accelerated rates of NAD reduction and oxidative phosphorylation [93]. Recent data support this notion of Ca^2+^-activated muscle oxidative phosphorylation cascade. It could be shown that Ca^2+^ increased the conductance of complex IV, complexes I + III, ATP production/transport, and fuel transport/dehydrogenases [94]. Ca^2+^ concentration has also been shown to directly stimulate ATP production through activation of the F_1_F_0_-ATP synthase at least in cardiac muscle [95]. Extrapolation of these data to the exercising muscle predicts a significant role of Ca^2+^ concentration in maintaining cellular energy homeostasis. The activation of the electron transport chain in mitochondria by Ca^2+^ concentration may significantly contribute to the Ca^2+^ stimulation of ATP production during exercise [94]. Functional relevance of Ca^2+^-dependent regulation of mitochondrial for exercise adaptation is revealed by observed improvement of mitochondrial Ca^2+^ homeostasis following an acute bout of prolonged eccentric exercise and may stabilize mitochondrial respiratory function [96]. A further indication of the relevance of mitochondrial Ca^2+^ content in human skeletal muscle is the increase of mitochondrial Ca^2+^ content after prolonged exhaustive exercise which is followed by a slightly increased state III and decreased state IV respiration. The restoration of the elevated mitochondrial Ca^2+^ level is slow and could be related to an increased state IV respiration, which together indicate uncoupled Ca^2+^ respiration during recovery [97]. In skeletal muscle, it seems that calcium dependent regulation of mitochondria is fiber type specific. It is shown that the sensitivity of the mitochondrial permeability transition pore to Ca^2+^-induced opening varies according to fiber type. The transient opening of the PTP is believed to play a physiological role in the regulation of mitochondrial (e.g., ΔΨ, ROS production, ion homeostasis) function [98]. Altogether, it is obvious that calcium is involved in the regulation of skeletal muscle mitochondiral respiration, although the mechanism explaining Ca^2+^-dependent mitochondrial regulation has to be further elucidated.

## 6. Ca^2+^ Ion Mediated Regulation of Muscle Plasticity

### 6.1. Ca^2+^ Ions and Skeletal Muscle Hypertrophy

Ca^2+^ ions also contribute to skeletal muscle hypertrophy as the adaptive response of skeletal muscle towards increased mechanical loading. The phosphatase calcineurin has been considered for a long time to contribute to skeletal muscle hypertrophy [99]. However, although the role of calcineurin for muscle growth in heart muscle is commonly accepted, its contribution to skeletal muscle growth is debated [100]. Calcineurin acts primary by dephosphorylation of the nuclear factor of activated T-cells [101]. While increased activation of calcineurin and NFAT isoforms *in vitro* have been shown to induce hypertrophy [99,102], *in vivo* results show skeletal muscle hypertrophy in slow but not in fast fibers [103] or, *vice versa*, Cyclosporine A (CsA)-induced inhibition of calcineurin did not block hypertrophy [100]. In other cases, calcineurin inhibition via CsA and mechanical stimulation did block hypertrophy of skeletal muscle suggesting a role for calcineurin in regulating mild hypertrophy [104]. Overall, those findings suggest an unclear and differential role of calcineurin in the mechanically-induced regulation of hypertrophy in fast and slow myofibers and myocardium [105]. However, calcineurin has been also shown to contribute to the control of skeletal muscle satellite cell differentiation via the regulated expression of MEF2, Myo-D and myogenin [106,107]. This important mechanism is vital for skeletal muscle regeneration of muscle fibers after injury [108] and essential for the long-term structural environment of hypertrophying skeletal muscle fibers [109]. Hence, besides a role for calcineurin in contributing directly to skeletal muscle hypertrophy it might be more linked to augment differential and redundant mechanisms that can enhance skeletal muscle growth under loading conditions in a more subtle manner.

### 6.2. Ca^2+^-Dependent Activation of Protein Degradation via Calpains

Calpains represent a family of non-lysosomal Ca^2+^-activated cysteine proteases. Skeletal muscle fibers contain different isoforms, the ubiquitously expressed µ-calpains and m-calpains, as well as the muscle specific isoform calpain-3 [110]. It still remains unclear which precise functions are mediated by calpains in skeletal muscle, but it is recognized that calpains are strictly involved in cell biological processes, such as differentiation, atrophy, and regeneration of skeletal muscles. Importantly, there is evidence that calpains are also involved in pathological skeletal muscle processes, e.g., muscular dystrophies, specifically limb-girdle muscular dystrophy type 2A [111].

A fine-tuned clarification of the regulation of this protease family in skeletal muscle tissue was suggested to be important for a deeper understanding of skeletal muscle protein turnover and muscle-damaging mechanisms [110]. Therefore, the authors aimed to investigate the localization and regulation of calpains in skeletal muscle tissue *in situ*. Under resting conditions, the Ca^2+^ concentrations in mammalian fast-twitch muscle fibers is around 20–50 nM and up to 250 nM, while a peak Ca^2+^ concentration during tetanic stimulation in murine muscle fibers of 1–2 µM was observed [112]. Baylor and Hollingworth [113] observed even 20 µM as transient peak Ca^2+^ concentration in stimulated muscle fibers. These data demonstrate that a precise determination of calpain localization and function is critical in order to understand their function, since local Ca^2+^ concentration seem to be highly variable. It was suggested by *in vitro* studies that µ-calpains require 3–50 µM free Ca^2+^ for half-maximal proteolytic activity, while m-calpains require 400–800 µM. Interestingly, these high Ca^2+^ concentration can be significantly reduced in the presence of phospholipids, small endogenous proteins or even specific substrates [114,115,116]. It has to be noted that Ca^2+^-induced activation of calpains is typically accompanied by autolysis of these enzymes. This processing step is highly important, since it reduces the Ca^2+^ concentration required for proteolytic activity significantly to values between 0.5 and 2 µM for µ-calpains and to 50–150 µM for m-calpains. Calpains have been associated with muscular dieases, such as limb-girdle muscular dystrophy type 2A [111] and Duchenne muscular dystrophy [117]. The latter work identified higher caplain activity states in muscle tissue from Duchenne muscular dystrophy patients, and an overexpression of calpastatins, inhibitors of calpain activity, were found to reduce dystrophic muscle phenotypes in mdx mice [118]. These data indicate clearly that calpains and, hence, the amount of free Ca^2+^, is a critical determinant in skeletal muscle function and specifically in skeletal muscle pathological circumstances.

When it comes to skeletal muscle regeneration, muscle satellite cells are a major source to regulate these processes. It has been proposed that calpains are involved in the regulation of satellite cell functions and, hence, play an important role in skeletal muscle regeneration. Raynaud *et al.* [119] observed that satellite cells express m-calpain in a cell cycle-dependent manner. In non-proliferating satellite cells entering the quiescent/G_0_ stage, m-calpain was found to primarily localize in the cytoplasm. However, after muscle injury, m-calpain is localized in satellite cell nucleus. When m-calpain was blocked by the specific calpain inhibitor MDL 28170, it was found that the satellite cells show defects in cell cycle stage regulation, prevention of Myo-D accumulation in the nucleus in the G_1_ phase and enhancement of Myf5 expression [119]. Furthermore, it was found that the calpain family of cysteine proteases is also involved in the regulation and remodeling of the cytoskeleton and the plasma membrane during satellite cell fusion events [120]. These data demonstrate that m-calpain/calpains are critical regulators of satellite cell function and are required for the initial skeletal muscle regeneration program after injury as well as for the tight remodeling and control of the satellite cell cytoskeleton and plasma membrane.

There is also evidence that calpains are regulated by physical exercise, as physical exercise changes/disturbs the resting Ca^2+^ concentration in skeletal muscle tissue. In this context, different authors [121] studied the effects of treadmill exercise in rats that took a soy protein isolate diet. Treadmill running transiently increased calpain activity in the gastrocnemius muscles, which was paralleled with an increase in creatine kinase activity. The applied soy diet reduced the running-induced activation of calpain, the fragmentation of myosin heavy chain (MHC), and the release of creatine kinase into plasma. The authors summarized that soy protein diet may be useful in preventing exercise-induced protein degradation in skeletal muscle. This may occur by inhibition of calpain-mediated proteolysis. However, the data available in the literature is still very limited, thus a sophisticated analysis of exercise-induced alterations of calpain activity cannot be reliably made, underscoring that there is still an important lack of knowledge about the influence of exercise on Ca^2+^ regulation which in turn stimulates the activation of calpains. This question should be addressed in future studies in order to shed light on these critical mechanisms fine-tuning protein turnover rates in skeletal muscle tissue.

### 6.3. Ca^2+^-Dependent Activation of Protein Synthesis

The major cellular pathway that regulates muscle growth via increased protein synthesis upon mechanical stimulation is the AKT-mTOR signaling pathway [122,123]. This molecular path was long believed to be preferentially activated via insulin-like growth factor-1 (IGF-1) and insulin. These factors bind to the IGF-1 receptor, which leads to the subsequent phosphorylation of protein kinase B (AKT), mammalian target of rapamycin (mTOR) and essential downstream targets (e.g., p70s6 kinase (p70s6k), ribosomal protein S6 (rpS6), eukaryotic elongation factor 2 (eEf2) and eukaryotic initiation factor 4E (eIF-4E) binding protein-1 (4E-BP1) that regulate translation [124,125]. However, it is meanwhile well known that especially mechanical stimulation of myofibers but also the increased availability of amino acids and Ca^2+^ ion influx within myofibers contribute to the multifactorial regulated activation of increased protein synthesis [126]. Recent work illustrated the complex role of mechanically-induced skeletal muscle hypertrophy, attributing to Ca^2+^-dependent processes an important role in the regulation of increased protein synthesis as response to mechanical loading. Besides RyR1, also stretch activated Ca^2+^ ion channels (SAC) contribute to the Ca^2+^ influx into myofibers which activates CaM [127]. CaM interacts with the human vacuolar protein sorting-34 (hVPS34) and activates mTORC-1 (mTOR-complex 1), importantly in the presence of essential amino acids [128]. It has been shown that Ca^2+^ ion availability in part depends on the activation of l-type amino acid transporters (LAT-1) [129]. These regulate the influx of leucine which co-activates together with CaM the hVPS34 protein and increases mTORC-1 signaling and muscle protein synthesis [99]. Indeed, during exercise, Ca^2+^-dependent signaling via calmoldulin kinase 2 was shown to inhibit translation initiation in skeletal muscle via selective inhibiton of 4E-BP1 and eEF2 [130]. The acute inhibition of signaling to translation during exercise is highly reasonable, as protein synthesis is an energy consuming process and its down regulation enables skeletal muscle to maintain energy levels to match the demands of acute contractile activity. Thus, Ca^2+^-dependent pathways contribute to maintain the cellular environment for increased protein synthesis and are able to modulate this process under conditions of energetic stress and during loading conditions.

### 6.4. Mitochondrial Adaptation via CaMK IV and PGC-1α

Ca^2+^ concentration has been shown to activate Ca^2+^/calmodulin-dependent protein kinase IV (CaMKIV). At the current stage, there is only minor information available about the role of CaMKIV in mitochondrial biogenesis and adaptation. It was suggested recently that an overexpression of the active form of CaMKVI results in skeletal muscle fiber type shifting from a fast-twitch to a slow-twitch fiber phenotype, indicating higher expression rates of type I and type IIa muscle fibers in these transgene mice. This slow-twitch fiber phenotype was accompanied by critical oxidative fiber type hallmarks, such as increased mitochondrial density and the expression of its master regulator PGC-1α [131,132]. The authors, hence, concluded that there is a direct link between CaMKIV and the mitochondrial regulator PGC-1α, which was further strengthened by the observation that PGC-1α is critically regulated by Ca^2+^ fluxes regarding its activity and expression rates [133]. To further elucidate the role CaMKIV in skeletal muscle tissue and specifically its involvement in skeletal muscle oxidative capacity regulation, Akimoto *et al.* [134] used a CaMKIV knockout mouse line described earlier [135]. The authors found, interestingly, that the CaMKVI knockout mice show a normal muscle fiber phenotype composition along with a normal mitochondrial enzyme expression in fast-twitch fibers. However, the slow-twitch soleus muscle showed an increase of slow-twitch type I myosin isoform by ~100%, indicating that CaMKIV is possibly involved in the transcription of type I myosin genes, specifically in slow-twitch muscles, such as the soleus. Interestingly, the increase in type I myosin protein in CaMKIV soleus muscle was not accompanied by an increase in PGC-1α expression [134], pointing to an uncoupling of these two proteins in the regulation of myosin heavy chain expression regulation and oxidative capacity adjustment in slow-twitch muscles. Furthermore, when subjected to chronic voluntary exercise training, the CaMKIV knockout mice developed a muscle fiber shift towards myosin IIa [134] indicating increased oxidative metabolic capacities induced by chronic exercise stimulations. These phenotypes did not differ to those observed in wild type littermates. Importantly, it has to be noted that the expression of CaMKIV in skeletal muscle tissue is still a matter of debate. While Zong *et al.* [136] found CaMKIV to be expressed in skeletal muscle tissue and to have a critical function as a master regulator of mitochondrial biogenesis, other authors [137,138] did not detect CaMKIV in skeletal muscle tissue of mice and also humans. Zong *et al.* [136] used a transgenic mouse line with a muscle-specific AMP-activated protein kinase (AMPK) deletion and substituted β-guanidinopropionic acid, a creatine analogue, to AMPK-deficient mice and to wildtype (WT) littermates. The authors found that CaMKIV is expressed in an AMPK-dependent manner, when WT mice were stimulated with β-guanidinopropionic acid and is accompanied by an increased expression rate of a second mitochondrial master regulator, known as PGC-1α. These data underscore firstly that CaMKIV seems to be involved in skeletal muscle mitochondrial regulation and secondly that the localization of CaMKIV in skeletal muscle is still under debate and, hence, its function has to be evaluated critically in the context of skeletal muscle adaptation potentials.

PGC-1α is, as mentioned above a master regulator of mitochondrial biogenesis and, thus, functions as a central fuel sensor expressed in skeletal muscles tissues [139]. In transgenic mice overexpressing PGC-1α, skeletal muscles were noted to have a higher number and density of mitochondria and thus a higher percentage of type I, oxidative, fibers [131,139]. According to these molecular observations, PGC-1α-overexpressing mice demonstrate higher endurance capacities caused by increased oxidative metabolism by efficient fatty acid substrate metabolism. In contrast to findings in the PGC-1α-overexpressing mice, transgenic mice carrying a muscle-specific deletion of PGC-1α show higher numbers of glycolytic muscles fibers, specifically type IIB and type IIX fibers accompanied by significantly reduced endurance exercise capacities compared to WT litter-mates [139].

It is known that the molecular characteristics of skeletal muscle fibers, such as the expression of distinct myosin heavy chains and subsequently, the metabolic cues of the muscle fibers, is critically regulated by the firing pattern of neurons at the α-motor neurons determining the innervations pattern and the ECC mechanism (see above) [140]. In this process, alterations in Ca^2+^ ion availability in the sarcoplasm are of critical importance (see below); however, in the context of PGC-1α regulation, Ca^2+^ ions also play central roles, thus determining metabolic characteristics of skeletal muscle fibers. How do Ca^2+^ ions induce PGC-1α regulations? It has been proposed that Ca^2+^ ions influence the activity of Ca^2+^-sensitive phosphatases and kinases, such as CaMK isoform II and IV. Furthermore, AMPK and also the mitogen-activated protein kinase p38 are critical regulators of PGC-1α activity, since these proteins have a direct influence on the activity of the PGC-1α protein [141,142]. Importantly, it has to be noted that physical exercise is able to regulate the amount and the activity of PGC-1α in a critical manner. In this context, Egan *et al.* [142] demonstrated that specifically short-term, but intense exercise, significantly increases PGC-1α mRNA in human skeletal muscle, when compared to moderate exercise regimes. The author also demonstrated that an increase in CaMKII activity due to increased Ca^2+^ fluxes in working skeletal muscles. The increase in CaMKII activity resulted in higher CREB phosphorylation levels in the nucleus that in turn resulted in a concerted manner with class IIa HDACs and ATF2 to higher PGC-1α promoter activities. These data demonstrate that physical exercise is an important regulator of PGC-1α abundance in skeletal muscle tissue that in turn depends on the Ca^2+^ flux characteristics reflecting the intensity of physical exercise [143,144].

## 7. Further Key Ca^2+^-Signaling in Skeletal Muscle

Skeletal muscle fiber type shifting represents a central capability of this specialized tissue to adapt to certain environmental cues, such as endurance exercise. The fiber type shifting is mainly regulated by the transcription factors nuclear factor of activated T-cells 1c (NFAT1c, [145,146]) and myocyte-specific enhancer factor 2C (MEF2C, [147]). Both transcription factors seem to be regulated by the Ca^2+^ concentration apparent in the sarcoplasm. There are excellent reviews addressing the NFAT- and MEF2C-dependent molecular mechanisms resulting in a changed skeletal muscle fiber type composition [22,148]. Therefore, we will not go into detail for these mechanisms.

Instead, we discuss the protein calcineurin (CaN) in more detail, since CaN is believed to contribute to skeletal muscle fiber shifting as well as NFAT and MEF2C do. CaN is a serine/threonine protein phosphatase that is under the direct control of Ca^2+^/calmodulin [149,150]. CaN is composed of a heterodimer bridging a calmodulin-binding catalytic subunit A and a Ca^2+^-binding regulatory subunit B [151]. An increase in cytoplasmic/sarcoplasmic Ca^2+^ concentration results in a complex formation consisting of Ca^2+^ concentration and calmodulin. These Ca^2+^-calmodulin complexes bear the capacity to activate CaN by binding to its regulatory subunit B. It was noted already decades ago that the deactivation time constant of calcineurin is quite fast [152]. Therefore, it was concluded that a sustained activation pattern of CaN is based on sustained elevations of cytoplasmic/sarcoplasmic Ca^2+^ concentration or cytoplasmic/sarcoplasmic Ca^2+^ transients that have to be exposed and generated at very short intervals [151].

The Ca^2+^ decoder CaN [151] has a profound effect on gene transcription and exerts its effects via modulations of the cytoplasmic-nuclear shuttling of a variety of transcription factors (e.g., NFAT or MEF2C). Interestingly, calcineurin is involved in the control of epigenetic modulatory enzymes, such as class IIa histone deacetylases (HDACs) that have been shown not only to regulate epigenetic mechanisms, but also, importantly, to be involved in the regulation of certain skeletal muscle fiber type programs [151]. These data highlight the importance of calcineurin in the determination of the skeletal muscle fiber phenotype and underline that Ca^2+^ transients are major causative modulators of skeletal muscle characteristics. However, it was observed that the shuttling of the mentioned transcription factors is much slower compared to a Ca^2+^-induced activation of CaN. Liu *et al.* [153] observed that the shuttling of NFAT from the cytoplasm to the nucleus occurs within some ten minutes. Accordingly, the shuttling of HDACs from the nucleus to the cytoplasm also occur this time frame [154]; however, he Ca^2+^ transients are much faster, occurring within seconds [151]. This discrepancy between the “coupled” factors, Ca^2+^ decoder CaN triggered by fast Ca^2+^ fluxes on the one hand, and relatively slow nucleo-cytoplasmic shuttling of skeletal muscle phenotype-regulating transcription factors on the other hand, implies that any sarcoplasmic Ca^2+^ concentration that is able to activate the transcription mechanism, not only has to be able to activate Ca^2+^ decoders, such as CaN, but also and importantly, has to maintain its activity for time intervals long enough for a significant translocation of the involved transcription factors [151].

Among the described evidence that calcineurin plays an important role in skeletal muscle phenotyping, there is still a strong debate about its role *in vivo*. Most data on CaN and its involvement in skeletal muscle fiber type shifting capabilities have been generated by inducing rather strong and unphysiological stimuli, including transgenic mice models, that do not reflect (patho)physiological conditions. For instance, Westerblad and colleagues [151] noted data from the literature leading to the assumption that even 4 h of exercise per day keep CaN activity at its basal activity levels for 20 h. Therefore, CaN activity is very low over the major part of a 24-h interval questioning its role in physiological skeletal muscle fiber type shifting capabilities. In this context, it was suggested [155] that a certain contraction threshold is evident *in vivo* resulting in a shift from glycolytic type II fibers towards oxidative type I fibers characterized by increased PGC-1α expression levels. According to these data, it still remains unclear what the main functions of CaN are *in vivo* under physiological conditions due to its very low activity that may not be sufficient to induce fiber type shifting *in vivo*. Thus, it has to be questioned whether CaN is a real modulator of skeletal muscle fiber type shifting phenotypes.

A recent paper built a connection between α-actinin-3 (Actn3) and calcineurin signaling in skeletal muscle tissue [156]. The *Actn3* gene encodes for the protein α-actinin-3 that is a major component of the skeletal muscle Z-disks in fast-twitch muscles [157]. Therefore, Actn3 is primarily observed in skeletal muscle tissue of sprinters or weight lifters underlining its association with skeletal muscle glycolytic capacities. Conversely, an Actn3 knockout in mice or the common human nonsense mutation R577X in the human *Actn3* gene results in skeletal muscle tissue that is characterized by increased endurance capacities, reduced muscle strength and increased mitochondrial metabolism capacities [158], suggesting why the human R577X mutations is frequently found in endurance athletes [159]. However, there is still a lack of knowledge about how the *Actn3* gene regulates the mechanical and metabolic capabilities of skeletal muscle fibers.

Now, there is new evidence that Actn3 is directly involved in the regulation of CaN activity [156]. The authors demonstrate using an Actn3 knockout mouse model that the activity of CaN is significantly increased in these muscles, priming the muscle towards an enduring and fatigue-resistance tissue. Interestingly, a second actinin isoform, Actn2, plays a significant role in this mechanism. Seto *et al.* [156] found that the α-actinin-2 protein is expressed in Actn3-deficient muscles and within these muscles Actn2 interacts with a key inhibitor of CaN, known as calsarcin-2 [156]. The higher binding affinity of Actn2 to calsarcin-2 compared to Actn3 is the decisive mechanisms, since the major CaN inhibitor calsarcin-2 is now unable to prevent CaN signaling. The increased activity of calcineurin now results in the activation of slow-twitch muscle-generating programs.

In summary, while there is still some inconsistency in the current literature, these data suggest a role of CaN signaling in the maintenance and regulation of skeletal muscle fiber characteristics. Also, it should be noted that proteins located within the skeletal muscle sarcomeric substructures, such as the α-actinins, critically involved in the regulation of CaN signaling and, thus, skeletal muscle activity and characteristics should be taken into consideration as well. Future studies should critically highlight these interactions in more detail, since it can be hypothesized that Actn3 is not the sole sarcomeric protein directly involved in calcineurin signaling.

## 8. Conclusions

Ca^2+^ ions are central molecules in function and plasticity of skeletal muscle. They have a multitude of tasks in the regulation of contraction, initiation of metabolism and a central role for activity-dependent adaptation of skeletal muscle via augmented Ca^2+^ signaling (Figure 1). The regulation of skeletal muscle function and adaptation by Ca^2+^ ions depends on the time course, amount and localization of Ca^2+^ in subcellular compartments of skeletal muscle. Ca^2+^ ions bind here to a multitude of target molecules which mediate the widespread role of Ca^2+^ by allowing their coordinate interplay for the acute and chronic regulation and adaptation of skeletal muscle.
